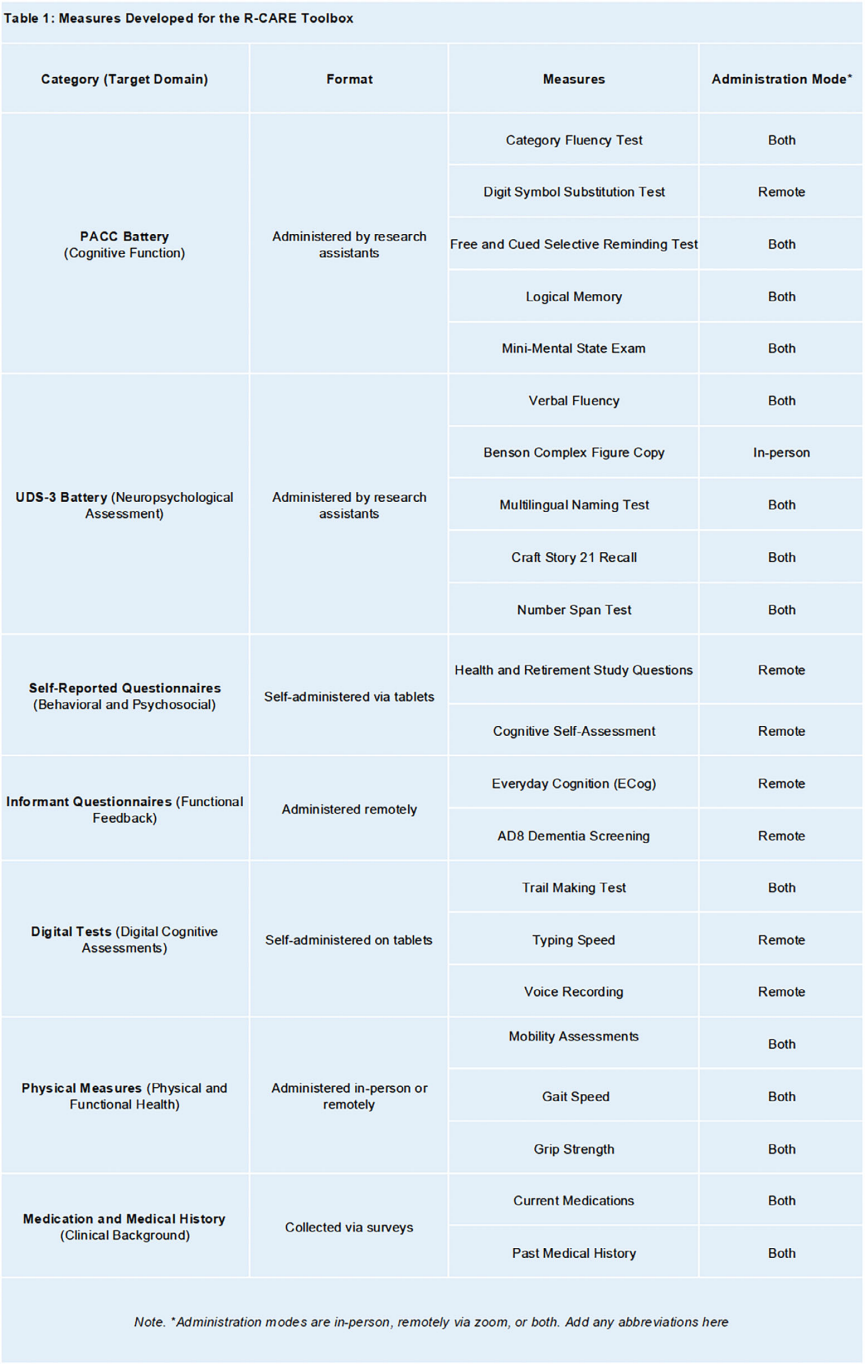# Open‐Source Development of The Remote Cognitive Aging and Alzheimer's Disease REsearch R‐CARE Toolbox for Advancing Remote Neuropsychological Assessment for Cohorts and Clinical Trials

**DOI:** 10.1002/alz70856_106978

**Published:** 2026-01-08

**Authors:** Hailey J. Andrews, Ali Ezzati, Laura A. Rabin, Nicole Sergeyev, Hannah Bodek, Jack D. Cameron, Chloe Moffitt, Robert Lavin, Richard B. Lipton, Nelson A. Roque

**Affiliations:** ^1^ The Pennsylvania State University, University Park, PA, USA; ^2^ University of California, Irvine, Irvine, CA, USA; ^3^ Brooklyn College of the City University of New York, Brooklyn, NY, USA; ^4^ Albert Einstein College of Medicine, Bronx, NY, USA

## Abstract

**Background:**

The Remote Cognitive Aging and Alzheimer's Disease REsearch (R‐CARE) Toolbox advances Alzheimer's disease and related dementias (ADRD) research by providing innovative, accessible, and standardized tools for remote neuropsychological assessments. R‐CARE addresses barriers including logistical challenges of in‐person assessments and limited access for underrepresented populations. By leveraging digital technologies, it adapts established assessments like the Preclinical Alzheimer's Cognitive Composite 5 (PACC‐5) and Uniform Data Set Version 3 (UDS v3.0) batteries for remote use.

**Method:**

The R‐CARE Team collaborated with clinical neuropsychology experts to adapt “gold standard” neuropsychological tests commonly used in ADRD trials and cohort studies for remote use. Tests unsuitable for direct remote implementation were modified or digitized to best maintain their key features. The toolbox incorporates measures from the UDS v3.0 and PACC‐5, physical measures, self‐ and informant questionnaires related to self‐perceived cognition, mood, functional capacity, social support, habits, and other relevant attitudes, experiences, and behaviors. The team programmed all instruments for delivery on the REDCap platform, and all digital cognitive assessments via the M2C2kit, supporting both in‐person and remote (videoconference) administration of tests. To promote open science and facilitate the adoption of digital and remote measures across the field, the R‐CARE Team aims to share all REDCap measures and digital measures via a publicly accessible resource‐sharing platform, GitHub, to allow for extension, translation, widespread use, and standardization.

**Result:**

The R‐CARE Toolbox includes 59 individual measures programmed for REDCap and 14 digital measures developed using the M2C2kit. These measures span cognitive, behavioral, physical, and functional health domains. A comprehensive list of these instruments is provided in Table 1. All instruments are shared here [https://github.com/rcare‐toolbox/redcap‐measures] to foster collaboration, support the standardization of in‐person and remote tests, and provide a foundational resource for further expansion by the wider research community.

**Conclusion:**

The R‐CARE Toolbox provides an open‐source solution for remote cognitive and functional assessment with the goal of improving accessibility, inclusivity, and scalability in ADRD research. By addressing logistical and sociodemographic challenges, the toolbox seeks to pave the way for innovative methodologies in clinical trials and cohort studies.